# Psychological Distance Toward Air Pollution and Purchase Intention for New Energy Vehicles: An Investigation in China

**DOI:** 10.3389/fpsyg.2021.569115

**Published:** 2021-04-01

**Authors:** Wenlong Liu, Lele Zeng, Qunwei Wang

**Affiliations:** ^1^College of Economics and Management, Nanjing University of Aeronautics & Astronautics, Nanjing, China; ^2^School of Management, Fudan University, Shanghai, China

**Keywords:** psychological distance, air pollution, risk perception, perceived price, purchase intention, new energy vehicle

## Abstract

Air pollution in China has been drawing considerable attention in recent years. The emergence of new energy vehicles (NEVs) provides hope to reduce air pollutant emission. However, consumers' recognition and acceptance of NEVs remain at the early stage. This research aims to explore how consumers' environmental concern influences their NEV purchase intention. Specifically, this research conducted an online survey and an experiment to address the following issues: (1) how consumers' psychological distance (PD) toward air pollution influences their purchase intention for NEVs, and does their risk perception of the consequences of air pollution mediate this influence; (2) whether consumers' perceived price level of NEVs plays a moderating role in the relationship between risk perception and purchase intention; and (3) whether the construal level of stimulus can be manipulated to influence consumers' PD toward air pollution to increase their purchase intention for NEVs. The results of study 1, based on a total of 293 valid samples, show that consumers' PD toward air pollution significantly affects their purchase intention for NEVs, and risk perception of the consequences of air pollution plays a considerable mediating role. Meanwhile, consumers' perceived price level of NEVs has a significant negative moderating effect on the relationship between risk perception and purchase intention. The results of study 2, based on an online experiment, show that the construal level of stimulus can influence consumers' PD toward air pollution, which in turn affects their purchase intention for NEVs. The findings of this research have implications for businesses' promotional strategies and governments' policies. For instance, low-construal-level promotional materials can be developed to arouse consumers' environmental concern, thereby facilitating their eco-friendly consumption behavior. Governmental financial assistance and other policies can also increase consumers' willingness to purchase NEVs.

## Highlights

- Consumers' psychological distance (PD) toward air pollution significantly affects their purchase intention for new energy vehicles (NEVs).
- Consumers' risk perception of the consequences of air pollution plays a considerable mediating role between their PD toward air pollution and their intention to purchase NEVs.
- Consumers' perceived price level of NEVs has a significant negative moderating effect on the relationship between risk perception of the consequences of air pollution and purchase intention.
- According to construal level theory and the experimental results of this research, low-construal-level stimuli can reduce consumers' PD toward air pollution.

## Introduction

Private vehicles in China have been increasing yearly along with the economic growth and the improvement of people's living standards. Despite the convenience they provide to our life, they have also caused many problems, such as continuous decline in air quality and increase in pollutant emissions, which are harmful to public health. Studies have shown that greenhouse gases and air pollutants resulting from the fuel consumption of traditional motor vehicles are among the main reasons for climate change (Wu et al., 2012). The emergence of new energy vehicles (NEVs) has brought hope to alleviate these environmental problems. NEVs are automobiles that use non-fossil fuels or partially use fossil fuels. Compared with traditional diesel locomotives, NEVs have a higher energy conversion rate, lower dependence on petroleum fuels, and lower emissions during driving (Wu et al., 2007). From the perspective of environmental protection, the promotion of NEVs is important in reducing air pollution problems, curbing global warming trends, and solving energy security issues. From the perspective of industry development, due to the importance of the automobile industry in China, strong policy support has been given to the development of NEVs, and industrialization and market promotion have good prospects. However, the quest for the NEV market just begun and most consumers have low recognition and acceptance of NEVs. The main barriers to the diffusion of NEVs are high prices, limited driving range and coverage of charging infrastructure, and long charging time, as well as the low level of knowledge of the NEV performances that consumers have (Williander and Stålstad, 2013; Cecere et al., 2018). Psychological approaches combined with the important contributions from the marketing literature can help identify consumers' attitudes toward NEVs and purchase intention (Sun and Morwitz, 2010; Arts et al., 2011; Cecere et al., 2018).

Scholars have introduced theories such as psychological distance (PD), construal level, and risk perception to understand individuals' environment-related attitudes and behaviors. PD usually refers to the perceiver's set of subjective or direct experiences of the distance from a stimulus (Trope and Liberman, 2010). PD can make individuals perceive an issue as less relevant and thus influence their behaviors. For example, farther PD toward environmental pollution may reduce consumers' intention to act environmentally friendly (Jäger and Weber, 2020). By contrast, closer PD toward environmental pollution can positively affect people's support and participation in environmental protection policies; PD toward environmental pollution can also affect people's choice of mode of transportation (Mir et al., 2016). On the basis of previous studies, consumers' PD from objects or risks can be changed by altering the construal level of stimulus (Trope and Liberman, 2003, 2010; Trope et al., 2007; White et al., 2011; Wang et al., 2019). Stimuli with low construal level are suggested to result in a higher concern for climate changes and higher willingness to act accordingly compared with stimuli with high construal level (Spence et al., 2012; Jones et al., 2017). Therefore, applying low construal stimulus is appropriate when communicating sustainable consumption issues toward consumers (Jäger and Weber, 2020). However, few studies have considered the relationship among construal level, PD toward air pollution, and the purchase intention for NEVs.

Risk perception can also explain people's psychology and behavior. For instance, studies have shown that investors' risk perceptions can drive market asset prices (Huber et al., 2019); risk perception of natural disasters can affect farmers' attitudes toward coping with risks (Hasibuan et al., 2020; and risk perception of climate change can affect environmental psychology and behavior (Bradley et al., 2020). The objects of risk perception are often diseases, natural disasters, safety accidents, or dangerous behaviors, but few studies consider risk perception of the consequences of air pollution. Risk perception can effectively influence consumers' purchase intention and behavior. Specifically, PD can affect individuals' risk perception of the consequence of climate change, which, in turn, influences their behaviors (Azadi et al., 2019).

Perceived price is always an important factor that affects consumer decisions (Lee et al., 2013; El-Said, 2020). Related research focuses on the difference between the effect of objective price and perceived price on consumers' purchase intention and the role of perceived price in purchasing decisions. For instance, some scholars have studied the impact of perceived prices and trust on purchasing behavior in online shopping (Kim et al., 2012). Customers' perceived prices for mobile services can significantly predict customers' price sensitivity levels (Liu and Lee, 2016). In view of sustainable consumption, high price may reduce consumers' intention to adopt environmentally friendly products (Best and Burke, 2018).

Based on the PD theory, this study explores the relationship between consumers' PD toward air pollution and their purchase intention for NEVs and verifies the mediating role of risk perception and the moderating role of perceived price level. The key problems solved in this study are as follows:

1. How consumers' PD toward air pollution influences their purchase intention for NEVs, and does their risk perception of the consequences of air pollution mediate this influence;
2. Whether perceived price level plays a moderating role in the relationship between risk perception of the consequences of air pollution and purchase intention; and
3. Whether the construal level of stimulus can be manipulated to influence consumers' PD toward air pollution, thereby affecting consumers' purchase intention for NEVs.

This research implemented two studies to address the abovementioned questions. The remaining sections of this paper are organized as follows. First, the literature on PD, the risk perception of the consequences of air pollution, and perceived price level is reviewed. The hypotheses of this research are proposed on the basis of the literature. Second, the measures used in the investigation and the data collection and analysis procedures of the two studies are introduced. The theoretical and practical implications are generated on the basis of our findings. In the section Conclusion, the limitations of this research and our future work are disclosed.

## Literature Review and Hypotheses Development

### PD and Purchase Intention for NEVs

PD and construal level theory are widely used to analyze consumer psychology and behavior and are important to predict and guide consumer behavior. PD is a person's perception of the distance between an object, a risk, or an event and the person him/herself, thereby affecting the person's motivation and preference for action (Trope et al., 2007). Objects are scattered in psychological space according to various dimensions, thereby forming different types of PD. Many discussions on the dimensions of PD have been conducted in academe. Among them, the most widely accepted theory is that PD includes four dimensions: temporal distance, spatial distance, social distance, and hypothetical distance (also called uncertainty) (Liberman et al., 2007; Liberman and Trope, 2008).

With regard to the correlation between PD and construal level, Mir et al. (2016) explored the influence of PD on people's choice of travel modes under different result frameworks. Chung and Park (2017) found that when a company's contradiction in social media is related to its morality, consumers who believe that the PD between them and the company is closer are more likely to conduct favorable evaluation of the company, and a similar pattern of purchase intention is observed. Azadi et al. (2019) studied the role of PD and risk perception in promoting farmers' adaptive behavior in climate change (Azadi et al., 2019). Loy and Spence (2020) found that reducing the PD from climate change can stimulate people's participation in environment-friendly behavior.

Construal level theory assumes that individuals perceive objects or topics as either rather concrete and detailed or abstract and holistic (Trope et al., 2007; Carmi and Kimhi, 2015). In essence, construal level refers to the differences of information expression and interpretation (Trope and Liberman, 2010). For instance, pictures are concrete representations that bear a physical resemblance to the referent objects, whereas words are abstract representations that carry the essence of the objects (Amit et al., 2009a,b). Therefore, words comprise a higher level of construal than do pictures. Construal level is one of the primary devices used to alter psychological distance (Soderberg et al., 2015). That is, more abstract (higher) construal will increase psychological distance, whereas more concrete (lower) construal will reduce psychological distance (Wang et al., 2019). Environmental pollution is generally described as an abstract topic and thereby as rather psychologically distant (Lorenzoni and Pidgeon, 2006; Jäger and Weber, 2020). However, previous studies have suggested that appropriate low construal stimuli might be an effective means for increasing environmentally friendly behavior to reduce individuals' PD to environmental pollution (Bashir et al., 2014; Jones et al., 2017). Air pollution is the most common form of environmental pollution worldwide. However, individuals generally perceive air pollution as a distant event, thinking that air pollution usually occurs far from their area (Bickerstaff, 2004). Using messages with low construal level may reduce people's PD to air pollution (Jäger and Weber, 2020). Reducing PD via communicating air pollution appropriately can increase individuals' willingness to be environmentally friendly, such as adopting a low-carbon transportation mode or purchasing green products (Mir et al., 2016; Jäger and Weber, 2020).

Based on the PD and construal level theory in past research, this paper proposes the following hypotheses:

H1. The closer consumers' PD is to air pollution, the stronger is their purchase intention for NEVs.
H2. Manipulating the construal level can affect consumers' PD, thereby influencing their purchase intention for NEVs. That is, the lower the construal level of the stimulus is, the closer the consumers' PD toward air pollution is and, thus, the stronger is their purchase intention for NEVs.

### Risk Perception of the Consequences of Air Pollution

Risk perception refers to an individual's feelings and understanding of various objective risks that exist in the outside world and emphasizes the effect of the individual's experience on intuitive perception and subjective experience (Starr, 1969; Slovic, 1987). Stone and Grønhaug (1993) proposes that the six-dimensional model of perceived risk is a widely used theoretical model, that is, perceived risk includes physical risk, financial risk, social risk, functional risk, temporal risk, and psychological risk. The study by Pu et al. (2019) on air pollution risk perception also divides it into six dimensions: risk benefit, environmental awareness, attention and knowledge, perceived risk, personal protection trust, and government control trust.

In related studies, most of the research objects are concentrated in fields such as natural disasters, diseases, safety accidents, and climate change. Ngo et al. (2017) found that personally participating in air quality research can improve the residents' awareness of the health risks of air pollution in informal settlements and help to increase their environmental awareness. Ban et al. (2019) explored health risk perception and its mediating role in heat wave protection behavior adaptation. Dinh et al. (2020) found that traffic risk perception is related to pedestrian safety behavior. Marshall (2020) believes that a complementary role exists between risk perception and safety culture. Xie et al. (2019) found that predictive factors of risk perception can also predict behavior and willingness. Castilho et al. (2015) studied consumer behaviors and factors that influence consumer satisfaction and risk perception of buying own brand food (Castilho et al., 2015). Lopes et al. (2020) use perceived social risk as a mediator to analyze the role of brand ethical issues in the purchase decision process. In the field of air pollution, PD to climate change can directly affect individuals' behavior and work indirectly through risk perception (Azadi et al., 2019). That is to say, if people perceive a close distance to air pollution, then they will have a high risk perception and thus behave environmentally friendly. Based on the above literatures, this study proposes the following hypotheses:

H3. The closer the consumers' PD toward air pollution, the higher their risk perception of the consequences of air pollution.
H4. Consumers' higher risk perception of the consequences of air pollution results in a stronger purchase intention for NEVs.
H5. Consumers' risk perception of the consequences of air pollution has a mediating role in the relationship between PD and purchase intention.

### Perceived Price Level

Price has always been considered as one of the important factors that affect consumer decision making. Consumers make different purchase decisions based on price perception and actual price comparison (Zeng et al., 2012). In general, the price is considered as the cost that consumers must pay to obtain the product or as the quality characteristics of the product itself.

Perceived prices are consumers' subjective feelings toward prices at a certain time (Kim et al., 2012). Jacoby and Olson (1997) distinguished between the objective price of a product and the consumer's psychologically encoded price. Compared with the objective price, the consumer's price coding of the product is relatively strict. For example, consumers compare the objective price of the product (the price set by the manufacturer) with reference prices (prices set by other manufacturers), and then the product price is coded to be higher or lower than the reference price; these results drive consumers' perception of the price while affecting consumer decisions (Jacoby and Olson, 1997). For NEVs, the perceived price can be defined as the consumer's perceived price level compared with those of traditional fuel vehicles.

Perceived price is often used to analyze and predict consumers' intentions and behaviors. Fecher et al. (2019) found that an unreasonable unit price would affect consumers' perception of price and purchase intention. Kim et al. (2012) studied the influence of perceived price and trust on purchasing behavior in online shopping. As a moderator, high price can weaken the effect of positive word of mouth on consumers' purchasing intention (El-Said, 2020). Specifically, despite the positive environmental result via adopting clean energy, such as solar and wind energies, the perceived price compared with carbon will reduce customers' intention to use (Best and Burke, 2018). Moreover, the decision making of purchase of NEVs is different from that of relatively low-cost green product. According to Diekmann and Preisendörfer (2003) and Cecere et al. (2018), when evaluating the purchase of relatively expensive goods, consumers will still attempt to optimize their utility while attributing a lower importance to environmental issues. For instance, an environmentally conscious consumer may easily reach a decision on paying $2 to buy a paper bag rather than to pay $1 to buy a plastic bag, but they may not that easily decide to buy a traditional fuel vehicle at $10,000 or to buy a NEV at $20,000. That is, despite the perception of the environmental risks, perceived price will play an important role in the adoption of NEVs. Similarly, according to Egbue and Long (2012) and Oliver and Rosen (2010), environmental risk perceptions influence the adoption of NEVs, but they are limited by the trade-off between environmental concerns and price. On the basis of these studies, we explore the moderating role of perceived price on the relationship between risk perception of air pollution and NEV purchase intention and propose the following hypothesis:

H6. Consumers' perceived price level for NEVs plays a moderating role in the relationship between their risk perception of the consequences of air pollution and purchase intention for NEVs.

## Research Design and Methodology

### Study 1: Relationship Between Consumers' PD, Their Risk Perception of the Consequences of Air Pollution, and Their Purchase Intention for NEVs

#### Measurement

**Figure 1:**
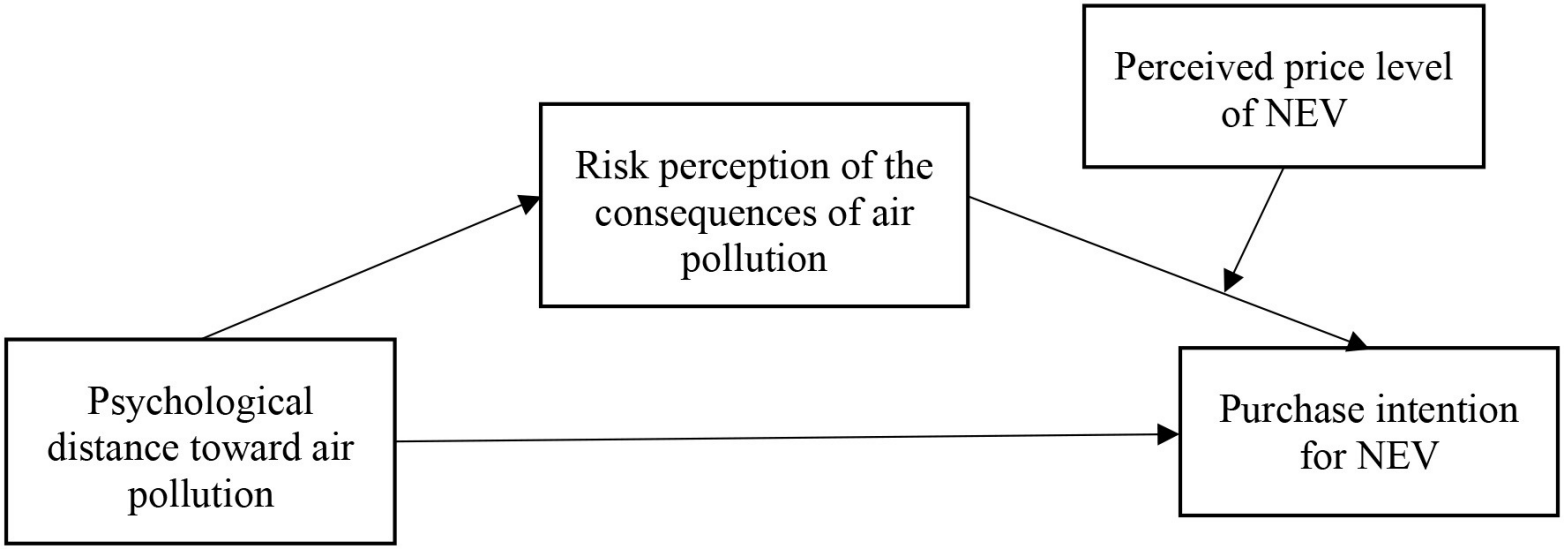
Conceptual model of study 1.

Figure 1 shows the conceptual model of study 1. To verify H1 and H3–H6, this study designed an online questionnaire (shown in Appendix 1) to investigate and measure the consumers' PD to air pollution, risk perception of the consequences of air pollution, perceived price level of NEVs, and purchase intention for NEVs, as well as to analyze and verify the relationship between different variables. The measurement of variables in this study is based on the scales used in related researches, combined with the actual context and the domestic environment, which are necessary to adjust and compile the final scale, and generate a formal questionnaire.

The questionnaire consists of five parts with a total of 32 questions. The first part presents the measurement of consumers' PD toward air pollution with a total of eight items. The items are derived from the work of Spence et al. (2012) and Wang et al. (2019) on the PD toward climate change. Consumers' PD toward air pollution is measured from four dimensions, namely, spatial distance, social distance, temporal distance, and uncertainty, using a five-point Likert scale. When measuring each dimension of PD, we used different scales and labels (positive and negative alternating approach) adopted from the study of Wang et al. (2019). For example, when measuring spatial distance, the participants were asked to rate statements, such as “My area may be affected by air pollution,” with 1 as strongly disagree (psychologically distant) and 5 as strongly agree (psychologically close), and “Air pollution mainly affects areas far away from me,” with 1 as strongly agree (psychologically distant) and 5 as strongly disagree (psychologically close). The second part presents the measurement of consumers' risk perception of the consequences of air pollution. The items are adopted from Fischhoff et al. (1978), Pu et al. (2019), and other studies on risk perception of technology and air pollution. Thirteen questions are asked about consumers' risk perception of the consequences of air pollution from six dimensions: risk benefit, environmental awareness, attention and knowledge, perceived risk, personal protection trust, and government control trust, using a five-point Likert scale. When measuring each dimension of risk perception, we used different scales and labels and handled them by using the same approach used in the first part. The third part presents the measurement of the perceived price level of new energy vehicles with a total of three items. The items are adopted from related studies by Kim et al. (2012) and El-Said (2020), also using a five-point Likert scale. The fourth part provides the measurement of consumers' purchase intention for NEVs, which consist of three items. The items are from Dodds et al. (1991), Cecere et al. (2018), and Armstrong et al. (2000). The last part presents a demographic variable that consists of five questions. Items of constructs are shown in Table 1.

**Table 1:** Items for PD, risk perception, and purchase intention.

**Variable**		**Item**	**Source**
PD toward air pollution	a. Spatial distance	a1. My area may be affected by air pollution.	Spence et al., 2012; Wang et al., 2019
		a2. Air pollution mainly affects areas far from me.	
	b. Uncertainty	b1. I am not sure if air pollution is indeed happening.	
		b2. The severity of the consequences of air pollution is exaggerated.	
	c. Social distance	c1. Air pollution mainly affects developed countries.	
		c2. Air pollution has a greater impact on me and my family.	
	d. Temporal distance	d1. Air pollution has already occurred or is happening.	
		d2. If anything, air pollution will occur in the very distant future.	
Risk perception of the consequences of air pollution	e. Attention and knowledge	e1. I am very interested in air pollution and want to learn more about past air pollution incidents.	Fischhoff et al., 1978; Pu et al., 2019
		e2. I often obtain information about air pollution through the Internet, TV, newspapers, and other media.	
	f. Perceived risk	f1. I know the causes of air pollution and their impact on health.	
		f2. Air pollution incidents that cause damage to the environment and human health occur from time to time.	
		f3. I am worried that air pollution will occur in the place where I live, causing damage to the environment and human health.	
	g. Government protection trust	g1. The government's environmental policy on air pollution control is trustworthy.	
		g2. The government provides the public with real information about air pollution.	
	h. Environmental awareness	h1. I am willing to reduce the use of private cars to protect air quality.	
		h2. I am willing to reduce the use of air-conditioner, elevators, microwave ovens, and other equipment to protect the air quality.	
	i. Personal protection trust	i1. I can rely on my own ability to avoid the harm caused by air pollution.i2. I have the knowledge to protect me from air pollution.	
	j. Risk benefit	j1. It is acceptable to sacrifice some air quality to develop the economy and increase people's income.	
		j2. Although some local pillar industries such as thermal power, steel, chemical, construction, and other industries cause serious pollution, they still need to exist.	
Perceived price level		m1. New energy vehicles are more expensive than traditional fuel vehicles. m2. Buying a traditional fuel car may enjoy a bigger discount than buying a new energy car. m3. The maintenance cost of new energy vehicles may be higher than that of traditional fuel vehicles.	Kim et al., 2012; El-Said, 2020
Purchase intention for new energy vehicles		n1. When you consider buying a car, consider how likely it is to buy a new energy vehicle. n2. When you decide to buy a car, how likely is it to choose a new energy vehicle? n3. How likely are you to recommend new energy vehicles to others?	Dodds et al., 1991; Armstrong et al., 2000; Cecere et al., 2018

#### Data Collection and Analysis

##### Descriptive Statistics

In this study, a total of 356 questionnaires were collected through an online-based survey from January 2020 to March 2020, among which 42 data were removed because the time spent on filling this questionnaire was <1 min. Another 21 responses were excluded because the participants already owned NEVs. Finally, 293 valid samples were used in the analysis. The demographic information distribution of the participants is shown in Table 2. Among the participants, 145 are males, accounting for 49.5% of the total samples; the other 148 are females, accounting for 50.5%. In terms of age, 106 participants are under 25 years old (36.2%), 135 participants are between 25 and 40 years old (46%), and the other 52 are over 40 years old. In terms of geographical distribution, the most participants are from East (*N* = 67) and Southwest (*N* = 49) China, and only 23 are from Northeast China. As for education level, most participants have undergraduate or higher educational background, accounting for 61.2%. In terms of income level, 75.4% of the participants have a monthly income below 10,000 RMB.

**Table 2:** Result of demographic statistics analysis (*N* = 293).

**Variable**	**Category**	**Number of people**	**Percentage (%)**
Gender	Male	145	49.5
	Female	148	50.5
Age	Under 25	106	36.2
	25–40 years old	135	46.0
	Over 40 years old	52	17.8
Area	East China	67	22.9
	North China	33	11.3
	Northeast China	23	7.9
	Central China	42	14.3
	South China	37	12.6
	Southwest China	49	16.7
	Northwest China	42	14.4
Education level	Below undergraduate	114	38.9
	Undergraduate	91	31.1
	Master's degree and above	88	30.1
Monthly income level	Under 5,000	142	48.5
	5,000–10,000	79	26.9
	Over 10,000	72	24.6

##### Measurement Model

In order to measure the proposed model, this study used SmartPLS 3.0 to test the reliability and validity based on the obtained data. PD and risk perception are modeled as second-order constructs with reflective–reflective approach: the first-order constructs are reflectively defined and the second-order constructs are also reflectively defined. As introduced in the section Measurement, consumers' PD toward air pollution includes four first-order constructs, namely, spatial distance, social distance, temporal distance, and uncertainty. Meanwhile, consumers' risk perception of the consequences of air pollution includes six first-order constructs, namely, risk benefit, environmental awareness, attention and knowledge, perceived risk, personal protection trust, and government protection trust. The reliabilities of all constructs were examined using two criteria: Cronbach's α and composite reliability (Straub et al., 2004). According to the results shown in Tables 3, 4, all the values of Cronbach's α and composite reliability are above the commonly acceptable level of 0.7.

**Table 3:** Summary of Cronbach's α of each construct (*N* = 293).

**Constructs**	**Number of items**	**Cronbach's α**
PD toward air pollution	8	0.916
Risk perception of the consequences	13	0.961
of air pollution		
Perceived price level	3	0.905
NEV purchase intention	3	0.925

**Table 4:** Results of convergent validity analysis.

	**Variable**	**Item**	**Factor loading**	**Composite reliability**	**AVE**	**Mean (standard deviation)**
PD	a. Spatial distance	a1	0.844	0.886	0.795	3.780 (1.010)
		a2	0.772			
	b. Uncertainty	b1	0.842	0.915	0.843	3.666 (1.021)
		b2	0.840			
	c. Social distance	c1	0.795	0.880	0.786	3.609 (1.028)
		c2	0.737			
	d. Temporal distance	d1	0.782	0.914	0.842	3.823 (0.983)
		d2	0.735			
Risk perception	e. Attention and knowledge	e1	0.853	0.919	0.850	3.672 (0.963)
		e2	0.828			
	f. Perceived risk	f1	0.852	0.934	0.824	3.618 (1.032)
		f2	0.841			
		f3	0.802			
	g. Government protection trust	g1	0.832	0.934	0.876	3.575 (1.004)
		g2	0.834			
	h. Environmental awareness	h1	0.816	0.930	0.868	3.684 (1.007)
		h2	0.838			
	i. Personal protection trust	i1	0.772	0.894	0.808	3.503 (0.904)
		i2	0.764			
	j. Risk benefit	j1	0.867	0.937	0.881	3.522 (1.008)
		j2	0.836			
Perceived price level		m1	0.916	0.940	0.840	2.830 (1.040)
		m2	0.907			
		m3	0.928			
Purchase intention		n1	0.940	0.953	0.870	3.710 (1.120)
		n2	0.923			
		n3	0.936			

Convergent validity was also examined using two criteria (Fornell and Larker, 1981): indicator loadings and average variance extracted (AVE). According to Table 4, all of the items exhibit a loading higher than 0.7 on their respective construct, and all the AVEs are also higher than 0.7, thereby satisfying both criteria of convergent validity. Meanwhile, the square root of AVE of each construct is greater than the correlations between the construct and all other constructs (shown in Table 5) in the model, demonstrating sufficient discriminant validity (Fornell and Larker, 1981).

**Table 5:** Correlations among constructs and the square root of AVE.

	**1**	**2**	**3**	**4**	**5**	**6**	**7**	**8**	**9**	**10**	**11**	**12**
Spatial distance	**0.892**											
Uncertainty	0.790	**0.918**										
Social distance	0.770	0.706	**0.886**									
Temporal distance	0.628	0.704	0.584	**0.918**								
Attention and knowledge	0.795	0.801	0.768	0.689	**0.922**							
Perceived risk	0.756	0.760	0.709	0.665	0.732	**0.899**						
Government control trust	0.752	0.797	0.712	0.703	0.824	0.725	**0.908**					
Environmental awareness	0.747	0.816	0.720	0.746	0.752	0.752	0.739	**0.936**				
Personal protection trust	0.782	0.785	0.747	0.764	0.795	0.706	0.733	0.802	**0.932**			
Risk benefit	0.727	0.780	0.741	0.719	0.794	0.725	0.821	0.764	0.762	**0.939**		
Perceived price level	−0.514	−0.50	−0.414	−0.371	−0.338	−0.379	−0.332	−0.360	−0.350	−0.361	**0.917**	
Purchase intention	0.876	0.887	0.809	0.764	0.837	0.799	0.817	0.817	0.825	0.830	−0.690	**0.933**

*Diagonal elements are the square root of AVEs*.

##### Structural Model

**Figure 2:**
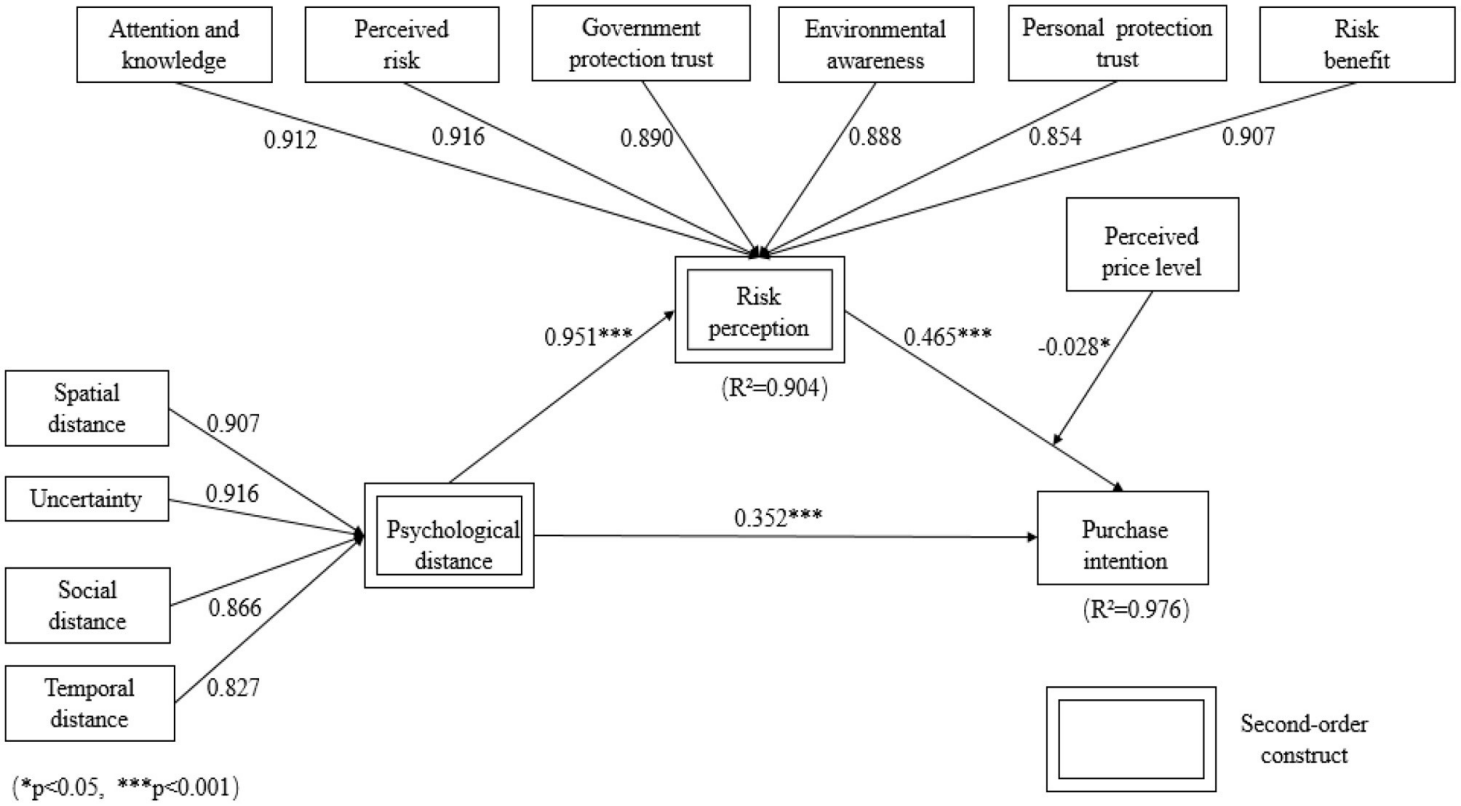
SEM analysis results of the structural model. Five-point Likert scales are used to measure constructs. The greater the values are, the closer the psychological distance, the higher the risk perception, and the stronger the purchase intention.

**Figure 3:**
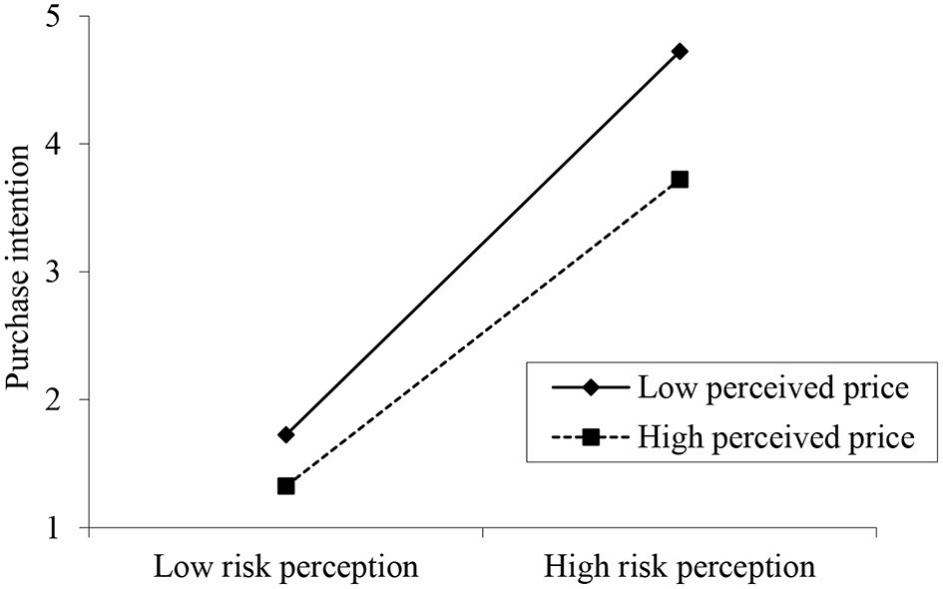
Moderating effect of perceived price level.

To test the structural model, a structural equation modeling (SEM) method based on SmartPLS 3.0 was used. As shown in Figure 2, all the factor loadings for reflective measures of the second-order constructs are >0.7. The path coefficient values shown in Figure 2 are exhibiting significant relationships among constructs with all *P*-values <0.05. Specifically, the closer the consumers' PD from air pollution, the higher intention they have to purchase NEV (β = 0.352, *p* < 0.001). Meanwhile, psychological closeness to air pollution will lead to high risk perception toward the consequences of air pollution (β = 0.951, *p* < 0.001). High risk perception eventually results in high NEV purchase intention (β = 0.465, *p* < 0.001). In accordance with Chin (1998), a bootstrapping with 5,000 resamples and 95% confidence interval was run to examine the mediating effect of risk perception toward the consequences of air pollution. Table 6 shows that there exists indirect effect between PD and NEV purchase intention via risk perception. Perceived price level moderates the relationship between risk perception of the consequences of air pollution and NEV purchase intention (β = −0.028, *p* < 0.05). As shown in Figure 3, perceived high price will weaken the effect of consumers' risk perception on their NEV purchase intention. According to the above results, H1 and H3–H6 are supported.

**Table 6:** Mediating effect of risk perception.

	**β**	**Standard deviation**	***t***	**Sig**.	**Lower pound**	**Upper bound**
PD → risk perception → purchase intention	0.441	0.028	15.574	0.000	0.386	0.497

*Five-point Likert scales are used to measure constructs. The greater the values are, the closer the PD, the higher the risk perception, and the stronger the purchase intention*.

### Study 2: PD Toward Air Pollution and Purchase Intention for NEVs Under Different Construal Levels

#### Experiment Design

The purpose of study 2 is to investigate whether consumers' PD toward air pollution can be influenced by manipulating the construal level, thereby influencing consumers' purchase intention for NEVs. The participants were assigned into two groups. After reading the materials with high and low construal levels of stimuli, respectively, the PD toward air pollution and purchase intention for NEVs were measured. Specifically, previous studies have shown that pictures are more specific/concrete than textual descriptions and have a lower construal level (Amit et al., 2009b). That is to say, objects that are closer are associated with a lower construal level (Fujita et al., 2008). Therefore, the participants in the high construal level group read a text related to air pollution from a report by the World Health Organization, which briefly introduced related information on global air pollution. The participants in the low construal level group watched a group of pictures related to air pollution corresponding to the text that the first group read. We borrowed pictures from news.ifeng.com and acquired permission from their office to use them in the experiment. As shown in Appendix 2, the question “By viewing the above text (pictures), I have a general/intuitive understanding of the current air pollution (1 for general and 7 for intuitive)” was used to test the manipulation of construal level (Trope and Liberman, 2010). The measurement scales and question items related to PD, risk perception, and purchase intention of NEVs are the same as those in study 1.

#### Data Collection and Analysis

##### Descriptive Statistics

In this study, a total of 132 valid samples were collected through a web-based experimental questionnaire survey. Specifically, 64 of the participants were assigned to the high-construal-level group, and the other 68 participants were assigned to the low-construal-level group. The demographic information distribution of participants is presented in Table 7, which shows that males and females account for 49.2 and 50.8% of the total samples, respectively. In terms of age, 32.6% of the participants (*N* = 43) are under 25 years old, 39.4% (*N* = 52) are between 25 and 40 years old, and the other 28% (*N* = 37) are over 40 years old. In terms of geographical distribution, the majority of the participants come from Northeast (*N* = 33), Southwest (*N* = 30), and East (*N* = 22) China, and only eight are from North China. In terms of education level, 68.9% of the participants have a bachelor or higher educational background. In terms of income level, participants with a monthly income of <10,000 RMB account for 83.3% of the total.

**Table 7:** Result of demographic statistics analysis (*N* = 132).

**Variable**	**Category**	**Number of people**	**Percentage**
Gender	Male	65	49.2
	Female	67	50.8
Age	Under 25	43	32.6
	25–40 years old	52	39.4
	Over 40 years old	37	28.0
Area	East China	22	16.7
	North China	8	6.1
	Northeast China	33	25.0
	Central China	12	9.1
	South China	14	10.6
	Southwest China	30	22.7
	Northwest China	13	9.8
Education level	Below undergraduate	41	31.1
	Undergraduate	71	53.8
	Master's or above	20	15.1
Monthly income level	Under 5,000	77	58.3
	5000–10,000	33	25.0
	Over 10,000	22	16.7

##### Construal Level Manipulation Test

To test the manipulation of the construal level, this study used SPSS 25 to conduct an independent sample *t*-test, with the dummy variable of the construal level (0 = high construal level, 1 = low construal level) as the grouping variable. As introduced in the experimental design, the lower the value is, the higher the construal level that participants perceive. The results shown in Table 8 indicate that group 0 perceives a higher construal level than group 1 does (*t* = −13.785, *p* < 0.001). Therefore, our manipulation is successful.

**Table 8:** Manipulation test result of the construal level.

**Test variable**	**Group**	**Cases**	**Mean**	**Standard deviation**	***t***	**Sig**.
Construal level	0	64	2.61	1.658	−13.785	0.000
	1	68	6.04	1.177		

##### Influence of Construal Level on PD Toward Air Pollution and NEV Purchase Intention

**Figure 4:**
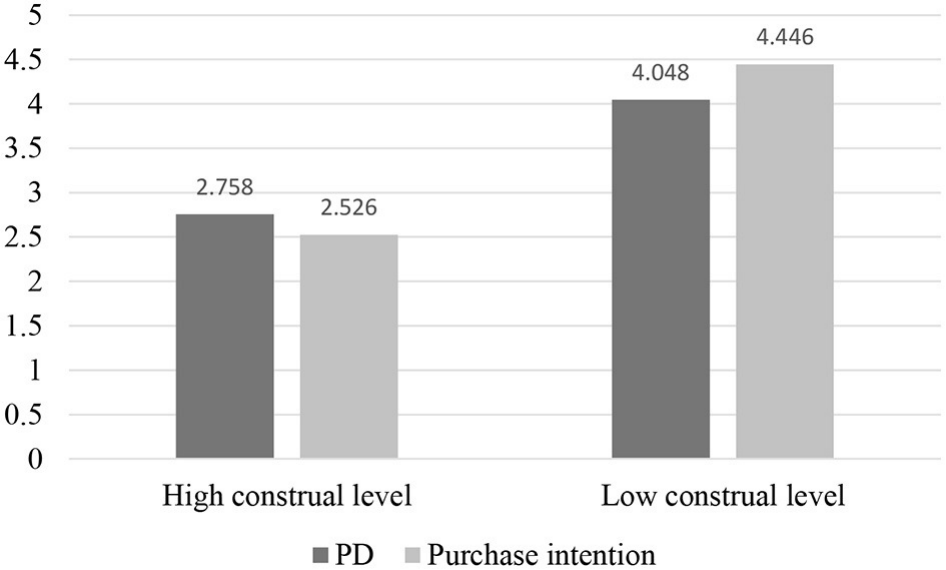
PD and purchase intention (PI) under different construal levels (CL). The greater the values are, the closer the PD and the stronger the purchase intention.

To verify H2, that is, whether different construal levels can affect the PD, thereby influencing consumers' purchase intention for NEVs, study 2 used SPSS 25 to conduct independent sample *t*-tests on the two sets of experimental data. The differences between the average PD of consumers toward air pollution (PD_highCL_ = 2.758, PD_lowCL_ = 4.048, *p* < 0.001) and the average purchase intention for NEVs (PI_highCL_ = 2.526, PI_lowCL_ = 4.446, *p* < 0.001) under different construal levels are shown in Table 9 and Figure 4, which show that different construal levels of stimulus in this experiment can significantly affect consumers' PD toward air pollution and NEV purchase intention. With considering the significant effect of PD on purchase intention in study 1 and the significant differences of PD and purchase intention caused by low and high construal level shown in Figure 4, it can be concluded that low construal level of stimulus can reduce consumers' PD toward air pollution, thereby enhancing their intention to purchase NEV. Therefore, H2 is supported.

**Table 9:** PD and purchase intention under different construal levels.

**Test variable**	**Group**	**Cases**	**Mean**	**Standard deviation**	***t***	**Sig**.
PD	0	64	2.758	0.853	−10.604	0.000
	1	68	4.048	0.513		
Purchase intention	0	64	2.526	1.0023	−12.974	0.000
	1	68	4.446	0.676		

*Five-point Likert scales are used to measure constructs. The greater the values are, the closer the PD and the stronger the purchase intention*.

## Discussion

In study 1, H1 and H3–H6 are verified. The results of the study indicate that the PD of consumers toward air pollution has a significant effect on the risk perception of consequences of air pollution and the purchase intention for NEVs. In other words, the closer the PD of consumers to air pollution, the stronger is the risk perception of the consequences of air pollution and the stronger is the purchase intention for NEVs. At the same time, the data results of study 1 also show that consumers' risk perception of the consequences of air pollution plays a mediating role in the relationship between PD and purchase intention. For H6, the data analysis results of study 1 verify that the perceived price level plays a significant negative moderating role in the relationship between risk perception of the consequences of air pollution and purchase intention, that is, if the consumer's perceived price level for NEVs is higher at this time, then the impact of risk perception on purchase intention is reduced. Study 2 has verified H2, which posits that according to the theory of construal level, manipulation of the construal level of stimulus can affect the PD, thereby affecting consumers' purchase intention for NEVs. That is, the higher the construal level is, the farther consumers' PD toward air pollution is and the weaker is their purchase intention for NEVs. In other words, consumers under a low construal level of stimuli, compared with a high construal level, have a closer PD toward air pollution and a stronger purchase intention for NEVs.

### Theoretical Implications

According to past research conclusions about PD, PD can explain consumers' wishes and decisions to a certain extent, and the PD toward environmental issues can affect consumers' attitudes to green products and purchase decisions (Spence et al., 2012). This study supports this view through empirical analysis. Consumers have a close PD toward air pollution, indicating that they have a stronger perception of air pollution and a higher level of environmental awareness, which in turn lead to a stronger purchase intention for NEVs.

Studies have shown that risk perception can effectively affect consumer behavior, and some researchers have verified that risk perception can play a mediating role between consumers' purchase intention and influencing factors (Castilho et al., 2015). In this study, consumers' PD of air pollution can be considered as influencing factors that affect consumers' purchase intention for NEVs, and the results of the study verify that the risk perception of the consequences of air pollution plays a mediating role between them, which is consistent with previous research theories. Furthermore, the construal level of stimulus is verified to be able to change consumers' PD, thereby influencing their purchase intention toward NEV. This finding provides a new perspective in studying consumers' PD toward environmental pollution and sustainable consumption-related decision making.

Previous studies on perceived price levels have shown that consumers make different purchase decisions by comparing perceived prices (Zeng et al., 2012). High perceived price levels tend to weaken consumers' purchase intention (Kim et al., 2012). The results of this study show that consumers' perceived price level of NEVs has a significant negative moderating effect on the relationship between risk perception of the consequences of air pollution and purchase intention. This result emphasizes the consideration of consumers' perceived price when studying their attitude and behavioral intention to environmentally friendly products.

### Practical Implications

The results of this study show that the closer the PD of consumers toward air pollution is, the greater is their cognition and feeling of air pollution, which then result in the stronger risk perception of the consequences of air pollution. The PD and risk perception will eventually influence their purchase intention for new energy vehicles. From a marketing point of view, reducing consumers' PD toward air pollution and improving consumers' risk perception of the consequences of air pollution should be considered among the main tasks in the promotion of NEVs. To realize these tasks, businesses should utilize mass media, such as websites, social media, and TV, rather than highly rely on promotional personnel. Businesses cannot only advertise their products but also exhibit stimuli with low construal level of air pollution in their commercial advertisements, such as pictures and videos showing the situation of air pollution, to arouse consumers' environmental concern, thereby improving their intention to purchase NEVs.

The research results show that the perceived price level has a negative moderating effect on risk perception of the consequences of air pollution and purchase intention. When promoting NEVs, marketers should highlight the advantages of the vehicles for their price, such as the energy saving of NEVs compared with that of traditional fuel vehicles; hence, consumers can perceive an acceptable price of NEVs. Meanwhile, NEV producer and retailers should provide warranties and guarantees for the high quality of after-sales service and disposal and recycling policies for used vehicles. This approach may help reduce consumers' perceived cost of purchasing NEVs, especially the non-monetary cost caused by the excellent service throughout the entire product life cycle.

Moreover, the results of this study also provide some implications for the government. Governments play an important role both by making regulations and by offering purchase incentives on the promotion of NEVs (Cecere et al., 2018). Especially, when making publicity materials related to environmental protection, low construal level stimulus, such as pictures or videos, rather than documents those with only words, should be adopted to reduce citizens' PD from environmental pollution and, in turn, motivate them to pursue environmentally friendly consumption behavior. Moreover, Oltra and Saint-Jean (2009) argue that market forces alone would provide insufficient incentives for environmental innovations and that consumers' willingness to pay for environmental improvements would be low. Specifically, the relatively high price of NEVs may, to some extent, weaken individuals' purchase intention. Accordingly, authorities should make policies to encourage citizens to purchase NEVs. For instance, governments should further improve their financial assistance and tax policy for eco-friendly product consumption and advertise this policy to ensure citizens' knowledge about it. The governments should also establish an entire society-scale reward system to facilitate citizens' environment-friendly behaviors.

## Conclusion

Based on the theory of PD and construal level, this study explores the relationship between consumers' PD toward air pollution and their purchase intention for NEVs. This study also verifies the mediating role of air pollution risk perception in this relationship. Based on the two studies, the following conclusions are drawn. First, consumers' PD toward air pollution has a significant effect on the purchase intention for NEVs. The risk perception of the consequences of air pollution can play a mediating role, that is, the closer is the PD of consumers toward air pollution, the higher is the risk perception of the consequences of air pollution and the stronger is the purchase intention for NEVs. Second, perceived price level has a significant negative moderating effect between risk perception of the consequences of air pollution and purchase intention. Third, when the construal level is manipulated, consumers' PD from air pollution can be affected, which in turn influences their purchase intention of NEVs. In other words, at a low construal level, consumers have a closer PD from air pollution and a stronger desire to buy NEVs.

Along with its contributions, this study has certain limitations. First, the high–low construal level grouping experiment in this study adopts the online experiment approach, so the degree of control over the experimental process needs to be strengthened. Moreover, picture and text presentations differ on various dimensions other than in their effect of construal level. For instance, either the font type or size of the text or the color or quality of the picture may affect individuals' understanding of the stimuli as well as the perception of the severity of environmental pollution. Thus, future work should explore more rigorous approaches to test the effect of construal level. Second, the sample sizes for the two studies are, to some extent, small. This condition may limit the significance of the results. In our next research, we will try to implement a relatively larger-scale investigation. Third, consumers' purchase intention for NEVs is affected by many factors, such as consumers' acceptance of related new technologies, available policy subsidies, and awareness of NEVs. This study only explored the influences of consumers' psychological factors, namely, PD and perceived risk of air pollution, on purchase intention. The effects of other factors remain to be further discussed. In our follow-up study, we will explore the interaction effect of consumers' intrinsic and extrinsic motivations on NEV purchasing intention. Specifically, we will first identify consumers' intrinsic motivators, such as PD to air pollution that was discussed in this research, environmental concern, and social responsibility, and extrinsic motivators, such as government policy, social norm, and promotional marketing. On this basis, we will examine what intrinsic and extrinsic motivators influence consumers' intention to purchase NEVs.

## Data Availability Statement

The raw data supporting the conclusions of this article will be made available by the authors, without undue reservation.

## Ethics Statement

The studies involving human participants were reviewed and approved by The Ethics Review Board of the College of Economics and Management of Nanjing University of Aeronautics & Astronautics. Written informed consent for participation was not required for this study in accordance with the national legislation and the institutional requirements. However, written informed consent was implied via completion of the questionnaire.

## Author Contributions

WL, LZ, and QW designed the study. LZ collected the data. WL and LZ analyzed the data and drafted the manuscript. All authors contributed to manuscript revision and read and approved the submitted version.

## Conflict of Interest

The authors declare that the research was conducted in the absence of any commercial or financial relationships that could be construed as a potential conflict of interest.
