# Supplementary material for: Psychological Distance Toward Air Pollution and Purchase Intention for New Energy Vehicles: An Investigation in China
Source: Front Psychol. 2021 Apr 1;12:569115. doi: 10.3389/fpsyg.2021.569115 (PMC8046919; doi:10.3389/fpsyg.2021.569115)
Supplement: Supplementary file 1 [file Presentation_1.pdf]

Appendix 1. Questionnaire for study 1

Study 1 Questionnaire

Hello!

We are conducting a survey on the relationship between consumers' PD to air pollution, risk perception, and purchase intention for new energy vehicles. The information you provide is only for research reference, and your private information will not be disclosed. Please consider your actual situation before selecting the answer. Thank you for your cooperation.

Please read the following statements, and judge whether each statement is in line with your thoughts according to actual conditions. 1 represents "highly disagree" and 5 represents "highly agree."

1. My area may be affected by air pollution.
2. Air pollution mainly affects areas far away from me.
3. I am not sure if air pollution is indeed happening.
4. The severity of the consequences of air pollution is exaggerated.
5. Air pollution will mainly affect developed countries.
6. Air pollution has a greater impact on me and my family.
7. Air pollution has already occurred or is happening.
8. If anything, air pollution will occur in the very distant future.
9. I am very interested in air pollution and want to learn more about past air pollution incidents.
10. I often obtain information about air pollution through the Internet, TV, newspapers, and other media.
11. I know the causes of air pollution and the impact on health.
12. Air pollution incidents that cause damage to the environment and human health occur from time to time.
13. I am worried that air pollution will occur in the place where I live, causing damage to the environment and human health.
14. The government's environmental policy on air pollution control is trustworthy.
15. The government provides the public with real information about air pollution.
16. I am willing to reduce the use of private cars to protect air quality.
17. I am willing to reduce the use of air conditioners, elevators, microwave ovens, and other equipment to protect the air quality.
18. I can rely on my own ability to avoid the harm caused by air pollution.
19. I have the knowledge to protect me from air pollution.
20. It is acceptable to sacrifice some air quality in order to develop economy and increase people's income.
21. Although some local pillar industries such as thermal power, steel, chemical, construction and other industries cause serious pollution, they still need to exist.
22. New energy vehicles is more expensive than traditional fuel vehicles.
23. Buying a traditional fuel car may enjoy a bigger discount than buying a new energy car.

24. The maintenance cost of new energy vehicles may be higher than that of traditional fuel vehicles.
25. When you consider buying a car, how likely you will consider to buy a new energy vehicle?
26. When you decide to buy a car, how likely you are to choose a new energy vehicle?
27. How likely are you to recommend new energy vehicles to others?
28. Your gender
29. Your province
30. Your age
31. Education level
32. Monthly income

Your answer has been submitted, thank you for your participation!

Appendix 2. Experimental survey questionnaire for study 2.

Hello!

We are doing a survey on the relationship between consumers' psychological distance to air pollution, risk perception, and purchase intention of new energy vehicles. The information you provide is only for research reference, and your personal privacy will never be disclosed. Please consider your actual situation before choosing. Thanks for your cooperation!

Please read/view the following information carefully:

Random material 1:

With regard to monitoring outdoor (environmental) air pollution around the world, most cities do not meet the safety level guidelines set by WHO, which exposes people to respiratory diseases and other health risks. In the cities studied, approximately half of the population is exposed to air pollution, which is many times higher than the level recommended by the WHO. These people face serious and long-term additional health risks. Some cities have sufficient data to compare the current situation with previous years, and most of them have increasingly serious air pollution. Many factors have contributed to this deterioration, including dependence on fossil fuels such as coal-fired power plants, dependence on private motor vehicles, inefficient use of energy in buildings, and use of organic fuel for cooking and heating (WHO, 2014).

Random material 2 :

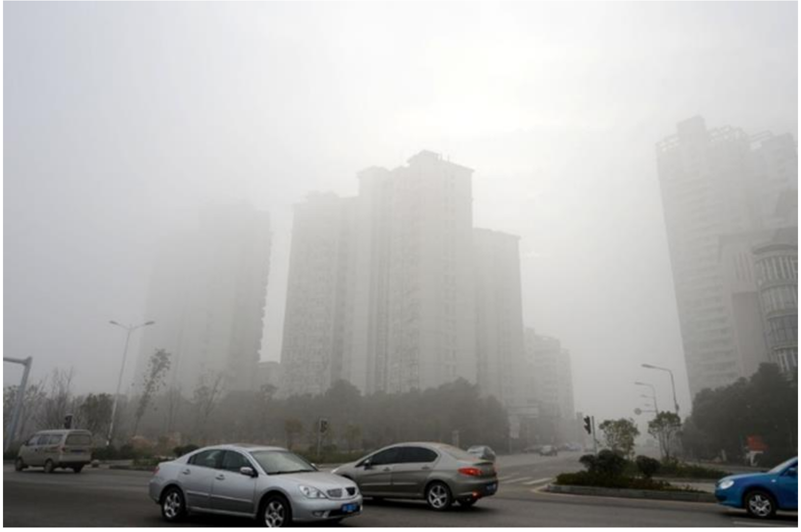

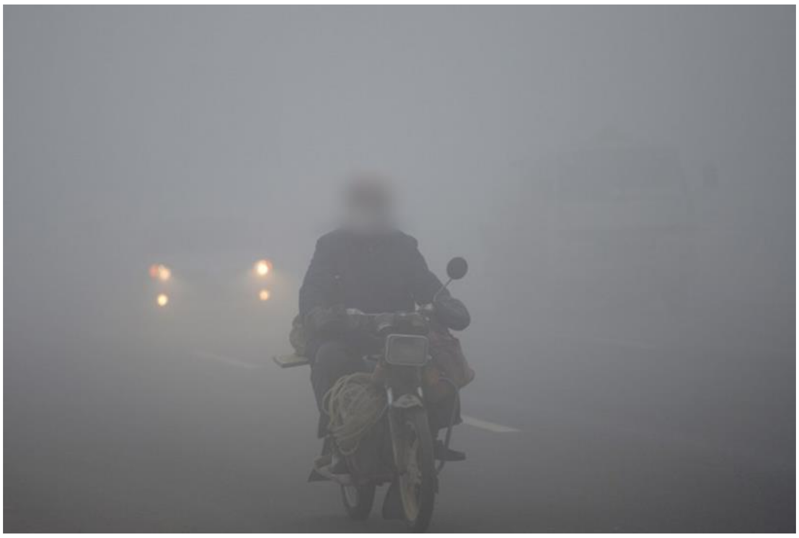

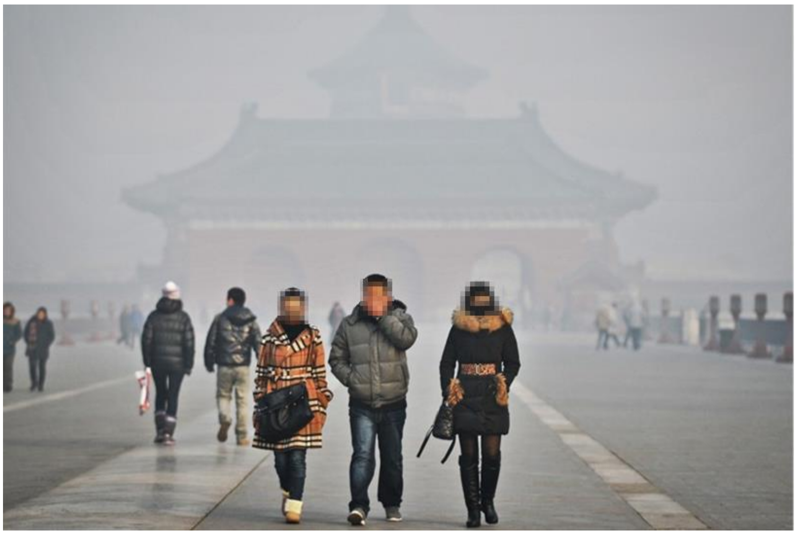

Notes: All the above pictures are borrowed from news.ifeng.com, china. The specific URL is http://news.ifeng.com/photo/hdnews/detail_2013_01/12/21123116_0.shtml#p=4. All identifiable human subjects in the pictures are processed with mosaic.

Manipulation question:

By reading (viewing) the above text (picture), I have a ____ understanding of the current air pollution (1 for general and -7 for intuitive).

Please read the following statements, and judge whether each of them is in line with your thoughts according to actual conditions. 1 represents “highly disagree” and 5 represents “highly agree.”

The questionnaire here is the same as it in Study 1.
